# Effect of hypercholesterolemia on circulating and cardiomyocyte-derived extracellular vesicles

**DOI:** 10.1038/s41598-024-62689-6

**Published:** 2024-05-26

**Authors:** Csenger Kovácsházi, Szabolcs Hambalkó, Nabil V. Sayour, Tamás G. Gergely, Gábor B. Brenner, Csilla Pelyhe, Dóra Kapui, Bennet Y. Weber, Alexander L. Hültenschmidt, Éva Pállinger, Edit I. Buzás, Ádám Zolcsák, Bálint Kiss, Tamás Bozó, Csilla Csányi, Nikolett Kósa, Miklós Kellermayer, Róbert Farkas, Gellért B. Karvaly, Kieran Wynne, David Matallanas, Péter Ferdinandy, Zoltán Giricz

**Affiliations:** 1 Department of Pharmacology and Pharmacotherapy, Semmelweis University, Budapest, Hungary; 2 Institute of Genetics, Cell- and Immunobiology, Semmelweis University, Budapest, Hungary; 3 ELKH-SE Translational Extracellular Vesicle Research Group, Budapest, Hungary; 4 HCEMM-SU Extracellular Vesicle Research Group, Budapest, Hungary; 5 Department of Biophysics and Radiation Biology, Semmelweis University, Budapest, Hungary; 6 Department of Laboratory Medicine, Laboratory of Mass Spectrometry and Separation Technology, Semmelweis University, Budapest, Hungary; 7 Systems Biology Ireland and School of Medicine, University College Dublin, Belfield, Dublin 4, Ireland; 8 Pharmahungary Group, Szeged, Hungary; 9 UCD Conway Institute of Biomolecular and Biomedical Research, University College Dublin, Belfield, Dublin 4, Ireland; 10 HUNREN-SE Biophysical Virology Research Group, Budapest, Hungary

**Keywords:** Exosome, Obesity, Dyslipidemia, Proteomics, Metabolomics, Inflammation, Membrane biophysics, Metabolomics, Proteomics, Extracellular signalling molecules, Lipid signalling, Stress signalling, Mechanisms of disease, Membrane trafficking, Protein transport, Inflammation, Proteomics, Biophysics, Biotechnology, Cell biology, Immunology, Molecular biology, Cardiology, Cardiovascular biology, Cardiovascular diseases, Dyslipidaemias, Metabolic disorders, Diseases, Cardiovascular diseases, Dyslipidaemias

## Abstract

Hypercholesterolemia (HC) induces, propagates and exacerbates cardiovascular diseases via various mechanisms that are yet not properly understood. Extracellular vesicles (EVs) are involved in the pathomechanism of these diseases. To understand how circulating or cardiac-derived EVs could affect myocardial functions, we analyzed the metabolomic profile of circulating EVs, and we performed an in-depth analysis of cardiomyocyte (CM)-derived EVs in HC. Circulating EVs were isolated with Vezics technology from male Wistar rats fed with high-cholesterol or control chow. AC16 human CMs were treated with Remembrane HC supplement and EVs were isolated from cell culture supernatant. The biophysical properties and the protein composition of CM EVs were analyzed. THP1-ASC-GFP cells were treated with CM EVs, and monocyte activation was measured. HC diet reduced the amount of certain phosphatidylcholines in circulating EVs, independently of their plasma level. HC treatment significantly increased EV secretion of CMs and greatly modified CM EV proteome, enriching several proteins involved in tissue remodeling. Regardless of the treatment, CM EVs did not induce the activation of THP1 monocytes. In conclusion, HC strongly affects the metabolome of circulating EVs and dysregulates CM EVs, which might contribute to HC-induced cardiac derangements.

## Introduction

Cardiovascular diseases (CVDs) are one of the primary causes of death worldwide^1^. Metabolic diseases, including hypercholesterolemia (HC), can induce, propagate and exacerbate CVDs via various mechanisms that are yet not properly understood. Extracellular vesicles (EV) are cell-secreted nano-sized membrane particles with various molecular cargo, including metabolites, proteins and nucleic acids^2^. EVs are involved in the pathomechanism of both CVDs and HC^3,4^. We have shown earlier that metabolic co-morbidities can ameliorate the innate stress response of the heart^5,6^, which is mediated by EVs^7^. Furthermore, one of the most common metabolic co-morbidities, type-II diabetes, can inhibit EV-mediated cardioprotection^8^. Besides, EVs of high-fat-fed animals can also exacerbate ischemic cardiac damage and induce cell death of cardiomyocytes (CM)^9,10^. These results suggest that EVs play a role in CVDs and HC.

EVs can regulate target cells via paracrine communication within a tissue or by getting into the circulation they can alter remote tissues. Therefore, to understand how HC play a role in CVDs via EVs, the analysis of both circulating and cardiac EVs is necessary. As of now, it is evidenced that the number of circulating EVs is increased in metabolic diseases^11–13^, including familiar hypercholesterolemia^14,15^, where their amount is associated with the risk of CVDs^16,17^. However, to understand the biological role of EV-related changes in HC, it is necessary to characterize the composition and content of EVs. The main constituents of EVs are membrane lipids, intravesicular and transmembrane proteins, nucleic acids and other metabolites. Changes in any of these constituents may result in altered biological responses. Barrachina et al. have analyzed the dysregulated protein composition of circulating EVs in obesity^18^, and a similar study also analyzed the miRNA cargo of EVs from obese patients^19^. However, how HC affects the metabolic profile of circulating EVs has not been assessed so far.

On the other hand, little is known about how HC affects the local EV communication of the heart. An earlier study presented that pathological secretion of endothelial-derived EVs in HC impairs cardiac angiogenesis^20^. CMs are the main constituent and the functional element of the myocardium. Their EV-mediated regulation of the cardiac milieu might have great importance in CVDs. However, the effect of HC on EVs released by CMs still needs to be addressed. Therefore, to understand the connection between CMDs and HC, an in-depth analysis of CM EVs is necessary.

One of the mechanisms that may connect CVDs and CMs and be regulated by EVs is inflammation. Earlier studies showed that CM EVs activate monocytes in various disease conditions, such as in angiotensin II-induced hypertrophy, thereby contributing to inflammation^21,22^. In contrast, in hypoxic conditions, EVs promote macrophage polarization to the reparative M2 phenotype^23,24^. These results suggest that CM EVs might play a role in HC-induced inflammation.

To fill the gaps and to extend our knowledge on the effect of HC on both systemic and cardiac EVs, we aimed to identify the metabolic alterations in circulating EVs and to analyze CM EVs in HC. Therefore, we isolated rat circulating EVs and compared their metabolomic changes to their plasma metabolome. In addition, for the first time, we assessed how HC affects the biophysical and biochemical properties of CM EVs and we analyzed their role in the activation of monocytes.

## Results

### Hypercholesterolemia alters metabolite composition of blood plasma-derived EVs

**Figure 1:**
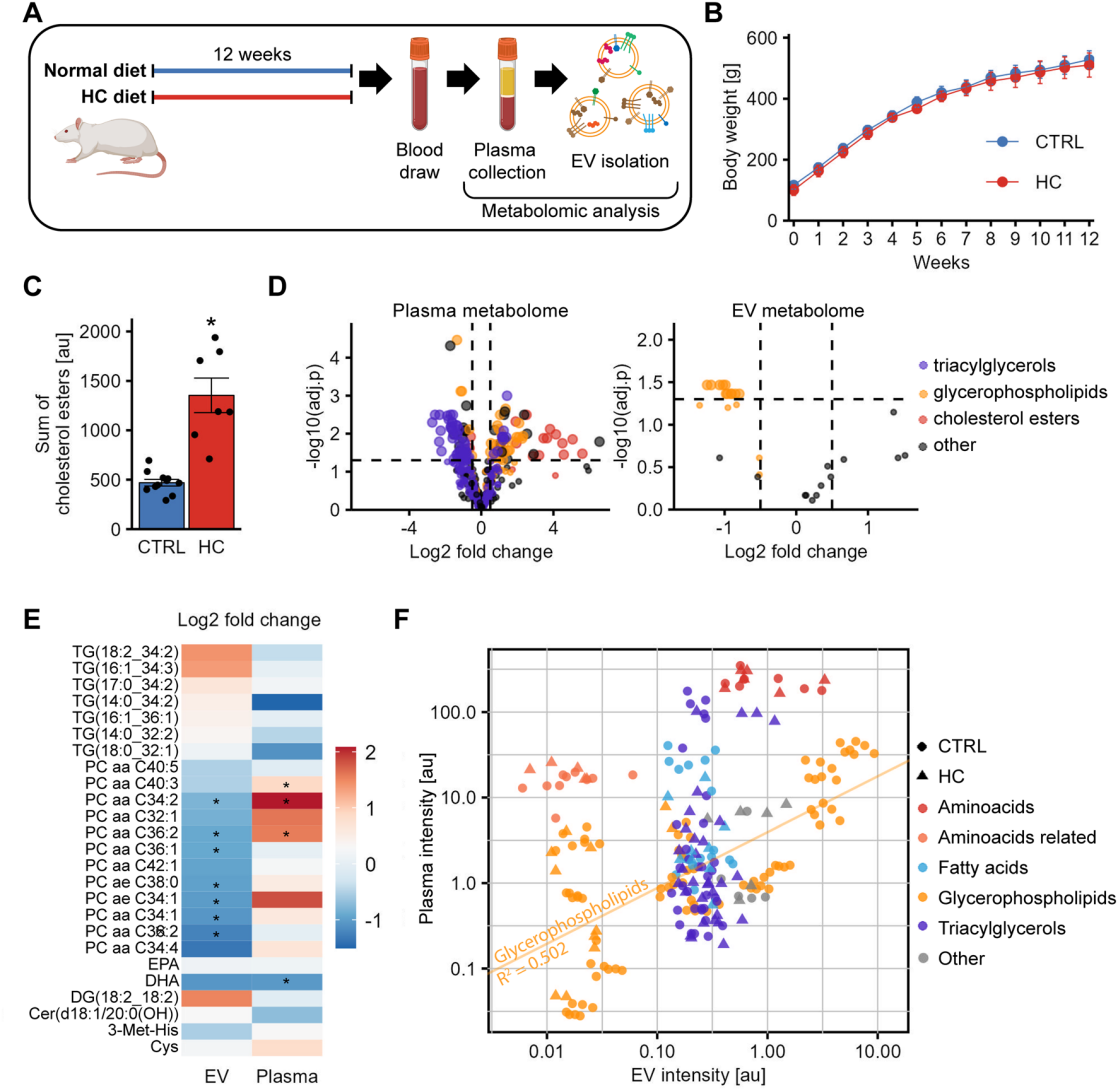
Metabolomic analysis of circulating EVs in HC-fed rats. (nctrl = 11, nhc = 7 for all experiments) (**A**) Schematic representation of the experimental plans. Male Wistar rats were kept on normal or HC diet for 12 weeks, then blood was collected and EVs were isolated from platelet-free plasma. Plasma and EV metabolome were analyzed. (**B**) Body weight of the animals. Diet did not affect body weight. (**C**) HC diet increased the amount of cholesterol esters in the blood. (**D**) Volcano plot of metabolome analysis on plasma (left) and EVs (right). In plasma, the intensity of numerous triacylglycerols was significantly reduced, meanwhile glycerophospholipids and cholesterol esters were enriched by HC. In EVs, the intensity of most glycerophospholipids detected was decreased by HC. (**E**) Heatmap for the metabolites detected both in EVs and in plasma. Multiple metabolites show opposite changes between EVs and plasma. (**F**) Linear correlation of multiple metabolites in EVs vs. plasma. Of the five metabolite groups analyzed, only glycerophospholipids showed a moderate correlation between plasma and EV intensities (see regression line, R2 = 0.502, p < 0.001). *p < 0.05 Student’s t-test CTRL vs HC; For metabolomics analysis (panel E) Benjamini–Hochberg false discovery p-adjustment was applied.

To extend our knowledge of how HC affects circulating EVs, we investigated how HC diet modifies the metabolite composition of circulating EVs and those of plasma (Fig. 1A). We have isolated EVs from rat blood using our protocol validated previously^25^, which results in non-detectable amount of common contaminants, such as Apolipoprotein A1. Of the 630 metabolites analyzed, 433 were detected in the plasma and only 29 in EVs (see Online Supplementary Material). The total amount of cholesterol esters identified was significantly elevated in the HC group (Fig. 1C) even though HC diet did not increase the body weight of the animals (Fig. 1B). In addition, plasma concentrations of several triacylglycerols (TG) were increased, whereas concentrations of several glycerophospholipids (GP) were decreased by HC diet (Fig. 1D, left panel). These results show that our model represents non-obese patients with hypercholesterolemia.

HC diet also altered the metabolite composition of EVs. The amount of several phosphatidylcholines (PCs) was reduced in EVs (Fig. 1D, right panel), whereas some showed opposite changes in plasma. Similarly, tendencies for inverse changes in TG levels between EV and plasma were observed (Fig. 1E). To further analyze the connection between plasma and plasma-derived EV metabolites, we correlated intensities of metabolites detected in both plasma and in EVs from the same animal. We found that only the amount of GPs was correlated between plasma and EVs, whereas there was no correlation between plasma and EVs in the concentration of amino acids, amino acid-related metabolites, fatty acids and TGs (Fig. 1F, Supplementary Figure S2). This analysis evidence that HC diet modulates metabolites in EVs and plasma differentially.

### HC increases EV release of AC16 CMs

**Figure 2:**
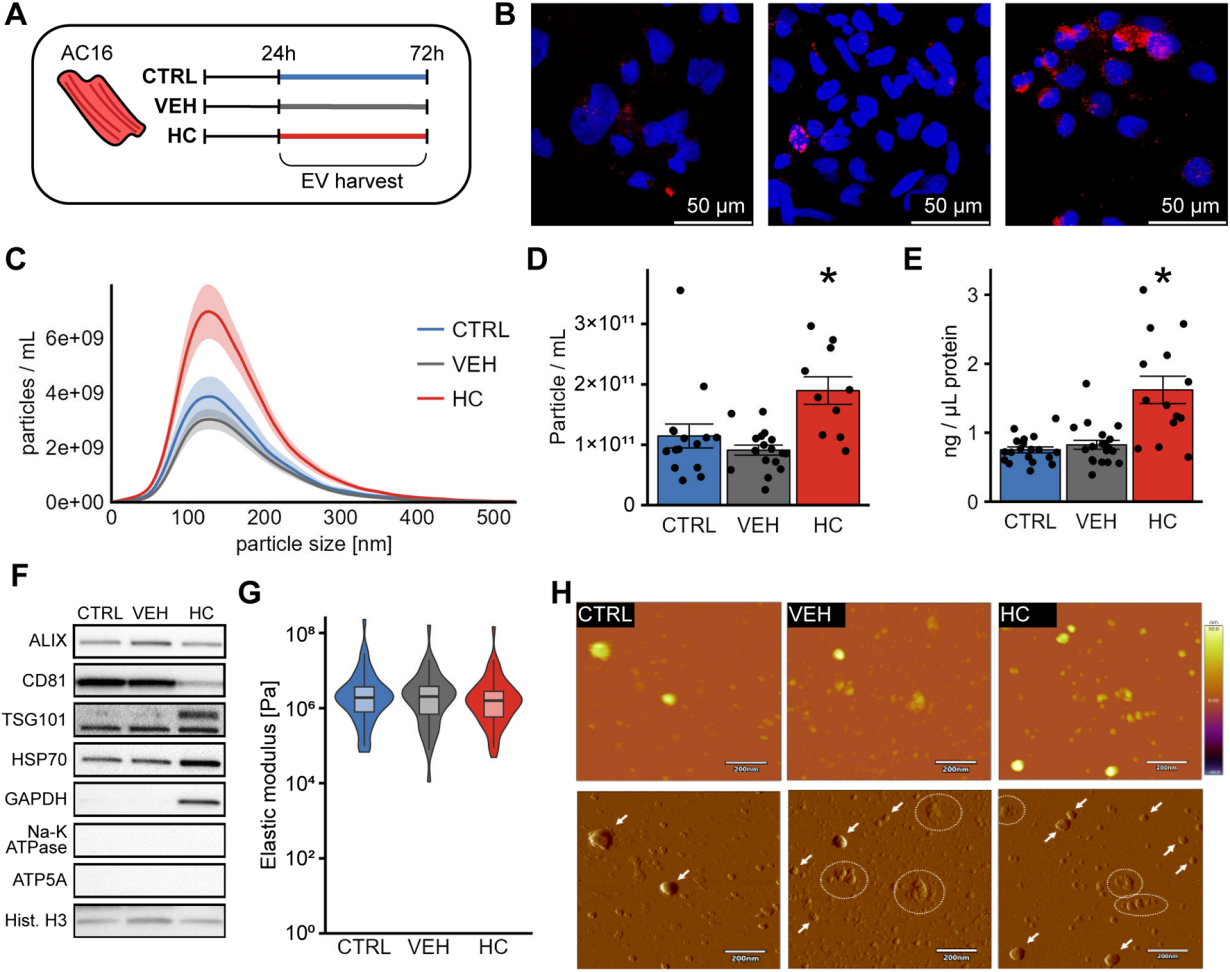
Analysis of EVs secreted by AC16 CMs. (**A**) Schematic representation of the experimental plans. AC16 human CMs were seeded for 24 h, and then the medium was replaced with serum-free medium with or without vehicle or HC supplement for another 48 h, then EVs were isolated from the cell culture supernatant. (**B**) Representative images of oil-red-o measurements on AC16 cells. Blue: DAPI (nuclei), red: Oil-red-O (lipid). Results show increased amounts of lipids upon HC treatment. (**C**) Size distribution of isolated EVs measured by NTA. Vesicles with diameters of 50–300 nm were detected in every experimental group. (nctrl = 15, nveh = 17, nhc = 10) (**D**) Particle number of EV isolates measured by NTA. HC increased the particle number significantly. (nctrl = 15, nveh = 17, nhc = 10) (**E**) Absolute protein concentration of the isolates measured with 280 nm light absorbance. HC increased the protein concentration significantly. (nctrl = 19, nveh = 20, nhc = 14) (**F**) Analysis of EV markers and potential contaminants using Western blot. Whole blots are presented in Supplementary Figure S3. (**G**) Elastic modulus of the isolates measured by AFM. No significant difference was observed. (nctrl = 58, nveh = 49, nhc = 54, out of at least three independent experiments) (**H**) Representative images of non-contact mode AFM measurements. First row: height-contrast, second row: amplitude-contrast images in the same view field. Arrows: EVs, Circles: Non-EV-like structures, with height < 10 nm. *p < 0.05 HC vs CTRL and VEH in ANOVA with Tukey’s post-hoc test.

To investigate the local effect of HC on cardiac EV, we next analyzed whether HC alters EV production of CMs using AC16 cells (Fig. 2A). Using Oil-red-o staining, we confirmed that the lipid content of CMs was increased due to treatment (Fig. 2B). When we analyzed the EV isolates, we found that HC treatment significantly elevated their particle (Fig. 2C and D) and protein concentration (Fig. 2E), suggesting that HC increased EV secretion of CMs. Besides, the size distribution of the isolates remained unchanged, according to NTA measurements (Fig. 2C, Supplementary Figure S3 A).

Our EV isolates were positive for EV markers Tumor susceptibility gene 101 (Tsg101) and ALG-2-interacting protein X (Alix), as well as transmembrane EV protein CD81. In addition, EV isolates contained glyceraldehyde-3-phosphate dehydrogenase (GAPDH) and heat shock protein 70 (Hsp70), which are common cargo proteins of EVs (Fig. 2F and Supplementary Figure S4). We tested potential contaminants, such as ATP synthase lipid-binding protein, Sodium Potassium ATPase and Histone H3, and we found that the samples contained Histone H3. (Fig. 2F and Supplementary Figure S4). We found significant differences in the particle/protein ratio (Supplementary Figure S3 B). These results confirm the EV origin of our membrane particles and that they might contain a certain level of non-EV material.

### HC treatment did not affect phosphatidylcholine content and elastic modulus of AC16 EVs

As we found that HC treatment modified the lipid composition of circulating EVs by decreasing the amount of certain PCs, we analyzed the total lipid and PC amount in AC16 EVs to unravel, whether similar changes can be observed in CM-derived EVs as well. We found that HC treatment did not change the lipid/protein ratio of the samples (Supplementary Figure S3 C). PC content, as normalized to the total lipid content, was also unaffected (Supplementary Figure S3 D). To further analyze the membrane properties of CM EVs, we applied AFM measurements, as cholesterol affects the rigidity, banding and elasticity of plasma membranes^26–28^.

Surface mapping revealed roughly circular particles with a partially flattened, curved surface that aligned well with the literature^29–32^. In addition, some associated vesicles and other smaller, rounded or multisegmented objects with a height lower than 10 nm were found, likely lipid- or protein aggregates or vesicle fragments (Fig. 2H). Based on AFM measurements, vesicles detected ranged from 10 to 300 nm in diameter with a median size of around 50 nm with a statistically significant difference between VEH vs CTRL or HC groups (Supplementary Figure S3 E), and vesicle height ranged from 10 to 100 nm with a statistically significant difference between HC vs CTRL or VEH groups (Supplementary Figure S3 F), however, the biological significance of these changes might be minor. Force spectroscopy was used to measure the elasticity of EVs. Elastic moduli were distributed between 10^5^ and 10^7^ Pa, which was in line with earlier data measured on liposomes and EVs^32–34^. HC treatment did not affect the elasticity of AC16 EVs (Fig. 2G). These results suggest that, in contrast to circulating EVs, HC does not affect the lipid composition of CM-derived EVs.

### AC16 EVs do not activate THP1 monocytes

**Figure 3:**
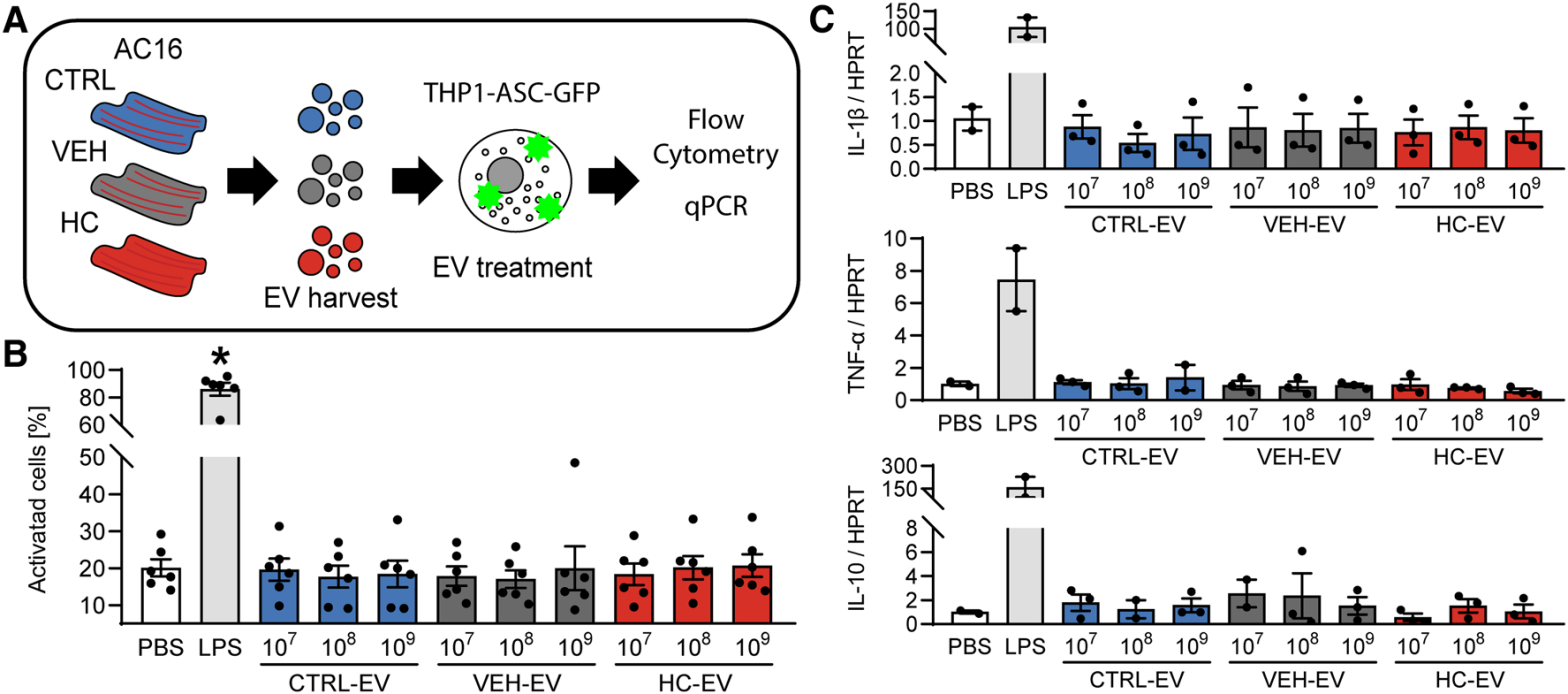
Analysis of immune cell activation of AC16 EVs using THP1-ASC-GFP. (**A**) Schematic representation of the experimental setup. EVs were isolated from AC16 cells and THP1-ASC-GFP cells were treated with the isolates, then flow cytometry measurement was applied to measure GFP expression and protein expression was analyzed with qPCR. (**B**) GFP expression measured by flow cytometry. LPS significantly increased the percentage of activated cells. EV treatment did not affect GFP expression, regardless of the dose or the experimental group (n = 6, all groups). (**C**) Gene expression analysis of IL-1β, TNF-α and IL-10 normalized to HPRT. No differences were observed (n = 2–3) *p < 0.05 vs PBS; ANOVA with Tukey’s post-hoc test.

As EVs play a role in inflammation and CM EVs modify immune cells in various diseased conditions^21–24^, we aimed to investigate the potential inflammatory effects of CM EVs in HC. We treated ASC-GFP-expressing THP1 monocytes with EVs derived from AC16 CMs in different doses. After 16 h of treatment, GFP expression in the monocytes was analyzed by flow cytometry to measure ASC-dependent inflammasome activation (Fig. 3A). We found that neither control nor HC EVs increased GFP expression in THP1-ASC-GFP cells (Fig. 3B, Supplementary Figure S5). We further analyzed the gene expression of pro-inflammatory factors Interleukin 1 beta (IL-1β), Tumor Necrosis Factor alpha (TNF-α) and anti-inflammatory factor Interleukin 10 (IL-10) in THP-1 cells, after treatment. No differential expression was observed in any of these genes (Fig. 3C). These results suggest that CM EVs do not activate monocytes.

### HC treatment modifies the protein composition of AC16 EVs

**Figure 4:**
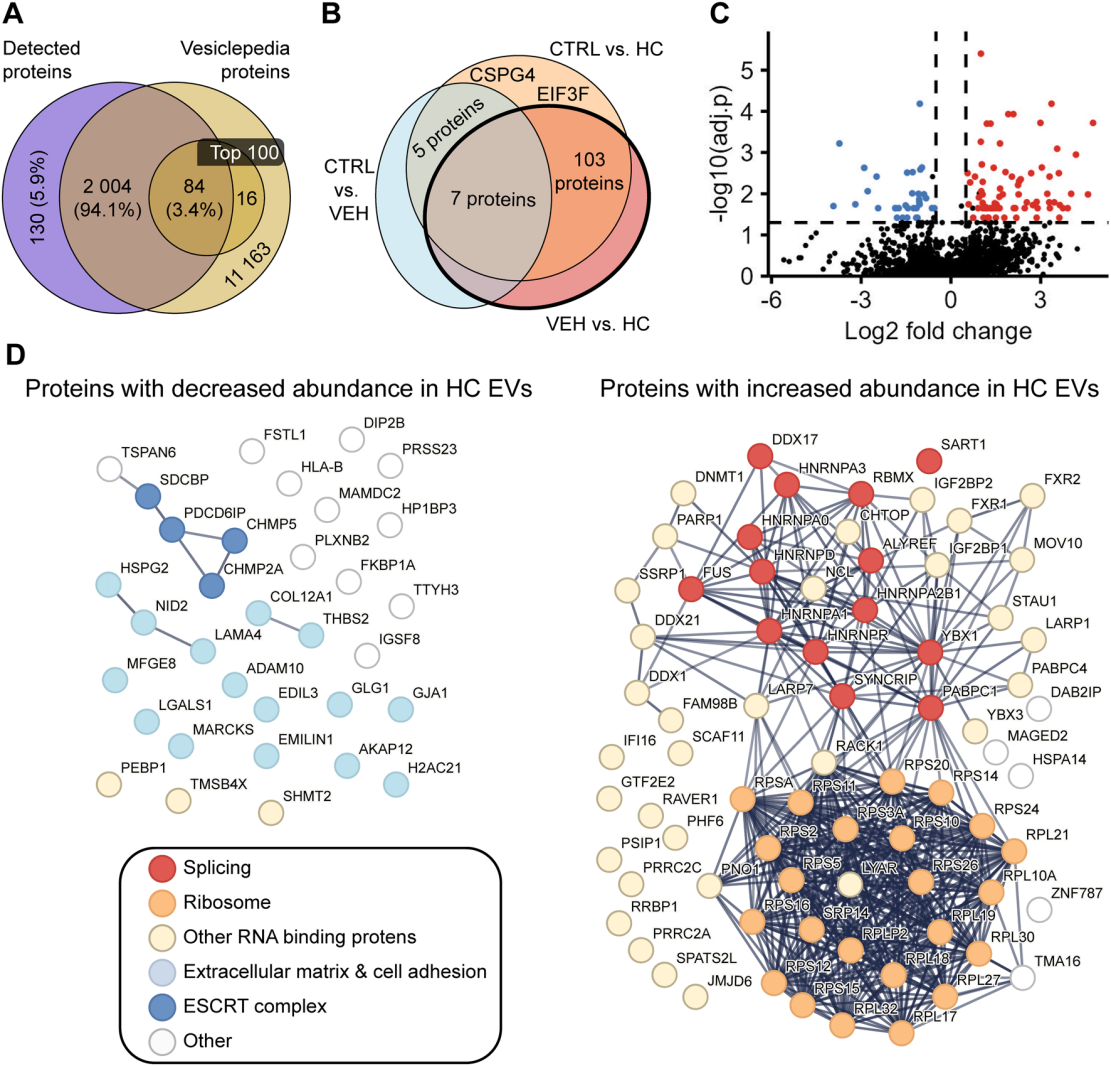
Proteomic analysis of AC16 EVs. (nctrl = 10, nveh = 10, nhc = 4, in all experiments) (**A**) Comparison of the proteins detected with the Vesiclepedia database. A total of 2137 proteins were identified of which 2088 were described in the database and 84 proteins were found among the 100 most frequently described EV proteins. (**B**) Venn diagram for the representation of the statistically significant differences between the experimental groups, using ANOVA followed by Turkey’s post-hoc test. (**C**) Volcano plot shows significant differences between the VEH and HC groups. (**D**) Interaction network of proteins with significantly different abundance between the VEH and HC groups. LEFT: Proteins with decreased abundance, RIGHT: Proteins with increased abundance.

The previous results refuted our original hypothesis that HC affects the inflammatory effect of CM EVs. In light of these results, we decided to analyze the protein composition of CM EVs isolated from HC-treated AC16 cells with liquid chromatography coupled with tandem mass spectrometry, to characterize the effect HC plays in the regulation of CM EV function. The data were quantified using label-free quantitation (LFQ). A total of 2235 proteins derived from 2218 genes were identified (see Online Supplementary Material). To perform a first quality control on our dataset, we compared these genes with the Vesiclepedia^35,36^ database. A total of 2088 genes overlapped with the database, of which 84 were among the 100 most frequently identified proteins in EVs (Fig. 4A). These observations reassured us of the quality of our isolation and the validity of MS-based proteomics to study EVs in our system.

To continue our analysis, we next compared the protein composition of EVs between the treatment groups using standard statistical analysis for proteomics datasets^37^. Thus, we performed ANOVA tests followed by Tukey’s post-hoc tests. We identified statistically significant differences in the amount of 117 proteins (Supplementary Table S4), of which, 12 showed differences between the CTRL and VEH groups. Five of these 12 proteins had significantly different abundance between the CTRL and HC, but not between the VEH and HC groups. Additionally, two proteins had significantly different abundance between the CTRL and HC but not between the VEH and HC groups. Finally, we found 110 proteins with significantly different abundance between the VEH and HC groups (Fig. 4B), which were selected for further analysis to explore proteomic changes induced by HC treatment in CM-derived EVs.

When we compared VEH and HC groups, we found that the abundance of 33 proteins decreased, while 77 proteins showed increased concentration in EVs upon HC treatment (Fig. 4C). Functional analysis of these proteins using gene ontology among the 33 proteins with significantly smaller amount, we identified numerous extracellular matrix (ECM) (GO: 0031012, FDR < 0.001) or cell adhesion-associated (GO: 0005925, FDR < 0.001) proteins. Remarkably, the amount of certain endosomal sorting complexes required for transport (ESCRT) constituents^38^ was reduced as well (Fig. 4D). Interestingly, most of the proteins with enriched concentration were associated with RNA binding (GO:0003723, FDR < 0.001) of which many proteins are part of the spliceosome complex or ribosomal complex. (Fig. 4D).

Altogether, our data show that HC treatment substantially affects the proteomic composition of CM EVs. Enrichment in RNA-associated proteins suggests that the RNA cargo of EVs can be modified as well. However, no specific cellular signalization pathway was identified that would be directly connected to HC-induced cardiomyopathy.

## Discussion

In the present study, we aimed to get a deeper insight into the impact of HC on circulating and CM EVs. We showed that HC diet reduced the amount of certain PCs in EVs and that changes in the lipid composition of the blood and EVs showed strikingly different patterns. Furthermore, we showed that HC treatment altered both the quantity and protein composition of EVs from CMs. Thus, contrary to our initial hypothesis, we show that HC does not affect THP-1 monocyte activation.

Previous works have characterized the proteomics and miRNA-omics of circulating EVs in obesity^18,19^. Besides, EV-transferred metabolites can affect remote tissues^39^, we therefore analyzed the metabolic profile of EVs isolated from HC-fed rats. In our isolates, we found that HC diet significantly reduced the amount of multiple PCs in circulating EVs. These results are in agreement with the studies by Blandin et al*.* who evidenced that total PC content was reduced in EVs isolated from adipocytes of high-fat diet fed mice^40^. Our results, together with miRNA-omics analysis of EVs from patients with metabolic disorders^19^, suggest that circulating EVs in HC are associated with a stress signal, as the amount of PC in EVs is associated with multiple disease states^41–43^. However, such changes could be originated from contaminants of the EV isolate, primarily from high-density lipoproteins, the fact that we have applied a well-established protocol that results in an EV isolate in which the markers of common contaminants, such as Apolipoprotein A1 are below the detection limit of western blot^25^, and that we identified an opposite directional change in lipoprotein-rich plasma, supports that the PC amount of the EV membrane or EV-associated, extravesicular constituents was decreased and such change is regulated independently of that of the plasma. However, for better validation, a more sensitive analysis of possible non-vesicular proteins, such as mass spectrometry could be implemented. As little is known about how the reduction of PCs affects EV-regulated mechanisms, functional experiments and comprehensive analysis of circulating EVs in HC, including how such EVs affect a healthy organism also need to be implemented. In conclusion, metabolic analysis of EVs may provide pathophysiological or diagnostic information on cardiometabolic derangements that might remain shrouded if plasma analysis was performed alone.

Importantly, our results, also shed light on how EV-mediated cardiac derangements can be derived from both circulating and cardiac tissue-derived EVs. We analyzed, how HC modulates CM EV secretion. As cardiac stress, such as altered Ca^2+^ concentration or hypoxia, is associated with elevated secretion of CM EVs^44–46^, our findings that CM EV secretion increases in HC may reflect the stress induced by the altered metabolic environment, similarly to circulating EVs. This is in line with previous observations as such stress response is suggested by Strauss et al*.*^47^, who found that treatment of Oli-Neu cells with cholesterol increased their EV secretion. This mechanism is proposed to eliminate cholesterol from the cells accumulated in the endosomal/lysosomal system in diseased conditions, such as in Niemann-Pick type C lipid storage disease. In addition, the increase in the number of EVs secreted may be the consequence of the higher amount of lipid rafts in CMs, as cholesterol plays a significant role in lipid raft formation^48^ and EV secretion correlates with the amount of lipid rafts^49^. Thus, our results together with previous findings show that cholesterol affects EV secretion through multiple mechanisms.

Importantly, we also show that modified EV secretory pathways may alter EV-associated protein composition as well. Our finding that the amount of certain ESCRT proteins decreased in HC EVs supports that HC-induced EV release in CMs may result from elevated cholesterol levels and increased lipid raft formation instead of an enhanced ESCRT-dependent EV secretory pathway. In addition, HC might affect the function of CM EVs in the maintenance of myocardial homeostasis, since the presence of multiple ECM and cell adhesion proteins was reduced in HC EVs or in their protein corona^50^. Furthermore, multiple proteins with tissue-remodeling functions were enriched in HC-treated AC16-EVs. Some of these are Connective tissue growth factor, also known as Cellular communication network factor 2 (CTGF/CCN2), which mediates fibrosis in cardiomyocytes and regulates proliferation, cell adhesion and angiogenesis^51^ or Heterogeneous nuclear ribonucleoprotein A1 (HNRNPA1), protein CYR61 and DNA (cytosine-5)-methyltransferase 1 (DNMT1) those can be found in cancer cell-derived EVs as well in which they play a role in EV-induced angiogenesis, proliferation, invasion or in vivo metastasis formation of recipient cells^52–54^. Besides, vesicular CYR61 is able to induce the expression of MMP-1 in remote cells as well^55^. In conclusion, these changes in the CM EV proteome may play a role in HC-induced cardiac remodeling.

Altered CM EV proteome also represents the physiological and metabolic status of their donor cells. Li et al*.* showed that the elevated amount of CYR61 in circulating EVs is associated with acute coronary syndrome^56^. Besides, the level of Gap junction alpha-1 protein, also known as Connexin-43 (GJA1, CX43) is decreased in circulating and CM EVs after myocardial infarction^57^. As the same changes in CYR61 and GJA1 were identified in HC-treated AC16 EVs, it may represent the stressed conditions of the donor cells. In addition, multiple proteins enriched in CM EVs after HC treatment, such as DNMT1, HNRNPA1, Double-stranded RNA-binding protein Staufen homolog 1 (STAU1) and Gamma-interferon-inducible protein 16 (IFI16) regulate the expression of various genes involved in lipid metabolism and adipocyte differentiation^58–61^. As CM EVs reach the circulation^62^ and establish communication between cardiac and adipose tissue^63–65^, these changes might aim to alleviate dyslipidemia and indicate the stressed metabolic condition of the CMs.

As most of the proteins that had elevated amounts in EVs after HC treatment bind RNA and some of these, such as GJA1, Nuclease-sensitive element-binding protein 1 (YBX1), Heterogeneous nuclear ribonucleoproteins A2/B1 (HNRNPA2B1) and HNRNPA1 play active role in RNA packaging to EVs^66–71^, we suggest that the RNA content of EVs is changed in HC as well, similarly to circulating EVs in obesity^19^. Also, complete 40S ribosomal subunits are presumably released in HC EVs, as the amount of most of its proteins was increased. However, the relevance of these changes needs further elucidation. In conclusion, our results indicate that HC treatment has a diverse effect on the molecular composition of CM EVs. Similar to circulating EVs, these changes outline a stress signal of the CMs propagated by EVs. To validate the suggested functional effects and to unravel whether these changes contribute to or compensate for HC-induced cardiac abnormalities, further functional experiments will need to be performed.

As EVs contribute to the physiological function of the immune system^72^, and since HC induces inflammation in multiple organs, including the heart and CM EV secretion is increased in inflammation^62^, it seemed plausible that EVs released from CMs in a HC environment may contribute to the activation of immune cells. However, our findings indicate that EVs derived from HC-treated CMs do not induce inflammasome-mediated activation of monocytes. This is also suggested by our proteomic analyses, which revealed a reduced abundance of the immunogenic protein HLA class I histocompatibility antigen, B-7 alpha chain (HLA-B). Of note, these findings are in contrast with earlier studies on circulating EVs from obese patients where an increased amount of various immunoglobulins and Complement C4-B protein was observed^18^, suggesting a pro-inflammatory phenotype. This contradiction indicates that CM- and circulating EVs might have opposing effects in the context of inflammation in HC. According to our results, as opposed to our original hypothesis, we concluded that CM EVs do not contribute to monocyte activation via inflammasome activation. However, further experiments may provide additional insights into the role of CM EVs in inflammation.

As we found that the amount of certain PCs was decreased in circulating EVs, we have analyzed the total PC content of CM EVs. Interestingly, the PC concentration in CM EVs was unchanged. Besides, the lipid to protein ratio and the membrane rigidity, defined by the membrane composition, remained unchanged in CM EVs as well. However, a complete comparison of the two EV sources, including both metabolomics on CM EVs and proteomics on circulating EVs may result in correlations between the sources, our results suggest that CM EVs are regulated differently in HC than circulating EVs.

In conclusion, here we broadened our knowledge of how HC alters the molecular composition of circulating EVs and thoroughly analyzed its effect on CM EVs. According to our results and earlier studies, EVs from both sources display a stressed condition. In addition, these EVs might contribute to the dysregulated cardiac homeostasis and tissue remodeling in HC. Further analysis of these EV-mediated pathophysiological mechanisms may improve our understanding of the interaction between cardiovascular and metabolic diseases.

## Materials and methods

### In vivo high cholesterol diet and blood collection

All procedures were approved by the National Scientific Ethical Committee on Animal Experimentation and the Semmelweis University’s Institutional Animal Care and Use Committee, and all experiments were performed in accordance with the ARRIVE guidelines. Male Wistar rats were fed either with standard (n = 11) or high cholesterol (n = 7) chow for 12 weeks. HC chow contained 2% cholesterol and 0.25% cholic acid. Animals were housed in a temperature (22 ± 2 °C)-, and humidity-controlled (50 ± 10%) room at a 12 h light/dark cycle and had free access to laboratory chow and drinking water ad libitum. After the 12 week period, the animals were anesthetized intraperitoneally with 60 mg/kg pentobarbital and their blood was collected from the abdominal aorta into Anticoagulant Citrate Dextrose-A vacuum tubes. Platelet-free plasma (PFP) was obtained by centrifugation twice with 2.500 × *g* at 4 °C, for 15 min as previously described^73^. PFP samples were stored at − 80 °C until processing (Fig. 1A). No animals were excluded from the study.

### EV isolation from the plasma

Plasma EVs were isolated and purified as previously described by Onódi et al.^25^. In brief, extracellular vesicle isolation was performed by iodixanol (60 w/V% iodixanol in ultrapure water; Axis-Shield, Oslo, Norway) density gradient ultracentrifugation (24 h, 120.000 × *g*, 4 °C). The EV-rich DGUC fractions (Supplementary Figure S1) were loaded into a HiScreen Capto Core 700 column (GE Healthcare Life Sciences) and size exclusion chromatography-based purification was performed. Vezics system (vezics.com) was used to implement the isolation.

### In vivo metabolomics

Metabolomic analysis of 0.01 mL EV- or PFP sample was performed using a Biocrates MxP Quant 500 kit (Biocrates AG, Innsbruck, Austria) according to the manufacturer’s instructions, as detailed in the Online Supplementary Material. The analytical procedure was guided by the laboratory information management software Biocrates MetIDQ (version 7.13.11, DB109-Nitrogen-2850 Revision 31.995, Base Version 109, Runtime version 1.8.0_181, Runtime architecture amd64; Biocrates A.G., Innsbruck, Austria). Analytical system control and data acquisition were accomplished using Sciex Analyst version 1.5.3. The analytical results were evaluated by MetIDQ and results were analyzed in R^74^. Metabolites were excluded from the analysis if less than half of the measurements (< 7 for CTRL and < 4 for VEH) were below the limit of detection (LOD) in both groups independently for EV and plasma samples. For the analytes included, values below the LOD were imputed with a normal distribution around half of the LOD. For correlation analysis, metabolite types for which at least twelve plasma-EV measurement pairs were available were analyzed with a linear regression model. For both linear regression and statistical analysis, data were log10 transformed.

### Culturing and HC treatment of AC16 cells

AC16 human cardiomyoblast cells from ATCC were cultured in Dulbecco’s Modified Eagle medium and Nutrient F12 1:1 mix (DMEM/F12) (Capricorn, Cat. no.: DMEM-12-A) supplemented with 12.5% heat-inactivated fetal bovine serum (FBS) (Corning, Cat. no.: 35-079-CV), 2 mM of L-glutamine (Corning, Cat. No.: 25-005.CI), 10 mM of HEPES (N-2-hydroxyethylpiperazine- N-2-ethane sulfonic acid) (Gibco, Cat. No: 15630-056) and 1% Antibiotic-Antimiotic Solution (Corning, Cat. No.: A5955-100ML). Cells were kept at 37 °C in a 5% CO2/95% air environment and subcultured by trypsinization (TrypLE) (Gibco, Cat. No.: 12604-021) when cells reached 90% of confluence. For HC treatment, cells were kept in medium supplemented with Refeed (Remembrane, Imola, Italy) hypercholesterolemic supplement or its vehicle (0.3% ethanol final concentration) for 48 h according to Makkos et al.^75^ (Fig. 2A). At the end of the treatment, cells were trypsinized and cell count was measured using a hemocytometer and viability was determined by Trypan blue (Cytiva, Cat. No.: SV3008401) staining. Cells were used only if viability reached 90%.

### Oil Red O staining

AC16 cells were seeded on CELLview cell culture microscope slides (Greiner Bio-One, Austria) at a concentration of 20,000 cells/well. After 24 h, the cells were treated with HC treatment solution, then cultured for another 48 h at 37 °C in a CO_2_ incubator. The cells were fixed in 10% neutrally buffered formalin, washed with ultrapure water and then with 60% isopropanol. The cells were then stained with Oil Red O solution (3 mg/mL) (Sigma, USA, Cat. No.: O0625-25G) for 10 min. This was followed by another wash with isopropanol followed by ultrapure water, and the nuclei were labeled with 1:1.000 dilution of DAPI (Cell Signaling Technologies, Danvers, MA, USA, Cat. No.: 4083S) for 1 min, and the samples were covered with Prolong Gold (Thermo Scientific, Waltham, MA, USA) mounting medium and coverslips were applied. Samples were examined with a Leica SP8 confocal microscope. Oil-Red-O dye was excited at 552 nm and the emitted fluorescence was detected in the range of 645–767 nm.

### Isolation of EV from AC16 cell culture supernatant

A total of 3.5 million AC16 cells were seeded in 175 cm^2^ culture dishes and 24 h later, the culture medium was changed to FBS-free medium. After 48 h of incubation, the culturing medium was collected in 50 mL centrifuge tubes and EVs were isolated using differential centrifugation. Cell supernatants were centrifuged at 300 × *g* at 4 °C for 10 min (Hettich Universal 320R; Rotor type: 1494 with Hettich 1427 adaptor). Then supernatants were centrifuged at 2,500 × *g* at 4 °C for 5 min (Hettich Universal 320R; Rotor type: 1494 with Hettich 1427 adaptor). The supernatants were transferred into 50 mL centrifuge tubes (Herolab Cat. no.: 253211) and centrifuged at 13,500 × *g* at 4 °C for 40 min (Hermle Z326K; Rotor type: 220.78 V20.). Next, they were transferred into ultracentrifuge tubes (Beckman Coulter, Cat. no.: 326823) and centrifuged at 174,900 × *g* at 4 °C for 3 h using an ultracentrifuge (Optima XPN-100; Rotor type: SW32Ti with adaptor 129.7). Pellets were resuspended in 120 µL phosphate-buffered saline (PBS) or 120 µL Tris-buffered saline (TBS) for atomic force microscopy (AFM). Protein concentration was measured with light absorbance at 280 nm by Implen N50 (Implen, München, Germany) nanophotometer.

### Nanoparticle tracking analysis

EV samples were analyzed by Zeta View PMX 110 (Particle Metrix, Meerbusch, Germany) nanoparticle tracking analysis (NTA) machine calibrated with 100 nm polystyrene beads according to the manufacturer’s protocol. Samples were diluted in PBS to a concentration of 50–300 particles in a view of sight. At least 1 mL of sample was injected into the machine and automated measurements were performed at 11 positions throughout the measurement cell, with two readouts at each position. Positions for which the software recommended exclusion were excluded from the final evaluation.

Instrument parameters were set as: temperature 25 °C, sensitivity 85, frame rate 30 frames per second and shutter speed 100. Post-acquisition parameters were set as: minimum brightness 20, minimum size 5 pixels and maximum size 1000 pixels. Results were multiplied by the dilution factor.

For size distribution plot, the mean of all the measurements was calculated, data were smoothed with Loess regression and visualized with ± SEM.

### Western-blot

For Western blotting, EV samples were lysed in radioimmunoprecipitation assay buffer (Cell Signaling Technologies, Danvers, MA, USA) supplemented with 1 mM of PMSF (Roche, Basel, Switzerland), and 0.1 mM of sodium fluoride, and complete protease inhibitor cocktail (Roche, Basel, Switzerland). Equal volumes of each sample were mixed with 1/4 volume of Laemmli buffer containing β-mercaptoethanol (Thermo Scientific, Waltham, MA, USA) and loaded on Tris–glycine sodium dodecyl sulfate–polyacrylamide gels (Bio-Rad, Hercules, CA, USA), and electrophoresed. Proteins were transferred onto a PVDF membrane (Bio-Rad, Hercules, CA, USA). Membranes were blocked in 5% bovine serum albumin (Bio-Rad, Hercules, CA, USA) in Tris-buffered saline containing 0.05% Tween-20 at room temperature for 2 h. Primary antibodies used were anti-TSG101 (ab83, Abcam, Cambridge, UK), anti-Alix (sc-53540, Santa Cruz Biotechnology, Dallas, Texas, USA), anti-CD81 (sc-166029, Santa Cruz Biotechnology, Dallas, Texas, USA), anti-albumin (sc-271605, Santa Cruz Biotechnology, Dallas, Texas, USA), Anti-ApoB (MABS2046, (Merck KGaA, Darmstadt, Germany), anti-fibrinogen (GTX54019, GeneTex, Irvine, Ca, USA) and anti-HSP70 (sc-66049, Santa Cruz Biotechnology, Dallas, Texas, USA). The presence of individual contaminating organelles was detected using the Organelle Detection Western Blot Cocktail (ab133989, Abcam, Cambridge, UK). The cocktail contained anti-sodium–potassium ATPase (plasma membrane), anti-ATP5A (mitochondria), anti-GAPDH (cytosol) and anti-Histone-H3 (nucleus) antibodies. Anti-mouse IgG, HRP-linked Antibody (7076s, Cell Signaling Technologies, Danvers, MA, USA) and Anti-rabbit IgG, HRP-linked Antibody (7074s, Cell Signaling Technologies, Danvers, MA, USA) secondary antibodies were used to detect the proteins. Signals were visualized using enhanced chemiluminescence kit (Bio-Rad, Hercules, CA, USA) using Chemidoc XRS + (Bio-Rad, Hercules, CA, USA) and analyzed with Image Lab software (Bio-Rad, Hercules, CA, USA).

### Determination of lipid and phosphatidylcholine content of EVs

Lipid content was determined as described previously by Visnovitz et al.^76^. In brief, 50 mg of vanillin (Sigma, W310727) was dissolved in 50 mL of 17% phosphoric acid (Sigma, 79617) to create phosphor-vanillin reagent. 200 µL of 96% sulfuric acid was added either to 40 µL of 1,2-Dioleoyl-sn-glycero-3-phosphocoline (DOPC) (Sigma, P6354) liposome standards, or to 40 µL of EVs suspended in sterile filtered PBS. After being vortexed, samples and standards were incubated at 90 °C in a fume hood for 20 min. Tubes were cooled down and 120 µL of phospho-vanillin reagent was added to each tube and 280 µL of each sample was transferred into a 96-well plate and was incubated at 37 °C for 1 h. Absorbance was determined at 540 nm using a plate reader (Multiskan Go, Thermo Scientific). Total PC content was measured using a colorimetric assay (CS0001, Merck KGaA, Darmstadt, Germany).

### AFM imaging and force spectroscopy of EVs

EV samples were diluted 100-fold with TBS buffer. One hundred µL of sample was deposited on freshly cleaved mica surface and incubated at 25 ± 1 °C for 30 min. Excess vesicles that did not adsorb on the substrate were removed by gently rinsing with ultrapure water, then 100 µL of TBS was added. These samples were examined using a Cypher ES atomic force microscope (Asylum Research, Santa Barbara, CA) using Olympus BL AC 40 TS cantilevers (nominal stiffness: 90 pN/nm, resonance frequency: 110 kHz, tip radius: 8 nm; Olympus, Japan) at 25 °C. Prior to measurements, cantilevers were calibrated by the thermal method in air^77^. Then, images were taken in non-contact mode, at 0.5–1 Hz line scanning frequency in buffer, oscillating the cantilever at its resonance frequency. Contact mode measurements were then performed for in situ force spectroscopy on selected spherical vesicles with topographical height exceeding 15 nm. During force spectroscopy, the cantilever was moved at speed of 1 µm/s from a pre-set height towards the vesicle until a load threshold of 100 pN was reached. It was then immediately retracted at the same speed. Deflection of the cantilever and thus force as a function of cantilever position (force-indentation curve or force curve) were recorded during the process.

Image and force curve analysis was implemented using the built-in algorithms of AFM driving software (IgorPro, WaveMetrics Inc., Lake Oswego, OR). Data were collected from a minimum of 5 individual experiments for each treatment group. Image analysis included only the particles with at least 10 nm height. Smaller particles were classified as non-vesicular structures and excluded from the analysis. EV diameter was determined as the diameter of a circle with an area equal to the surface area of the vesicle projected to the substrate. Vesicle height is defined as the difference between the highest point of the vesicle and the substrate. Young modulus of vesicles was obtained by fitting force-vesicle indentation curves from 0 (contact point) to 100 pN loading force with the modified Hertz model^78^.

### Mass spectrometry of EV proteins

AC16 EV isolates were vacuum-dried and transferred on dry ice to UCD Conway Institute Mass Spectrometry Resource. Samples were then resuspended in 50 µL of 50 mM Tris HCl (Thermo Scientific, Waltham, MA, USA), sonicated, and protein concentration was measured using a bicinchoninic acid (BCA) assay kit (Thermo Scientific, Waltham, MA, USA). Samples were normalized to protein concentration and dissolved in 6M Urea (Thermo Scientific, Waltham, MA, USA). The samples were then reduced by adding 8 mM of dithiothreitol (Merck KGaA, Darmstadt, Germany) and stirred at 30 °C at 1000 rpm for 30 min. Samples were then carboxylated by adding 20 mM of iodoacetamide (Merck KGaA, Darmstadt, Germany) and stirred at 30 °C at 1000 rpm for 30 min. Next, samples were diluted with 50 mM of Tris HCL to reduce urea concentration below 2 M. Samples were digested by Trypsin (Promega (Corporation, Madison, WI, USA) at 37 °C with 1000 rpm in 1:30 enzyme:sample ratio overnight. Digestion was terminated by adding formic acid (Thermo Scientific, Waltham, MA, USA) to 1% final concentration. Samples were purified on Empore^™^ Solid Phase Extraction membranes (Merck KGaA, Darmstadt, Germany), eluted in 60% acetonitrile and 0.1% Trifluoroacetic acid, vacuum dried and in 0.1% formic acid and each sample was loaded onto an Evosep tip (Evosep Biosystems, Buchwaldsgade, Denmark). The Evosep tips were placed in position on the Evosep One, in a 96-tip box. The autosampler is configured to pick up each tip, elute and separate the peptides using a set chromatography method (30 samples a day)^79^. The mass spectrometer was operated in positive ion mode with a capillary voltage of 1.700 V, dry gas flow of 3 l/min and a dry temperature of 180 °C. All data were acquired with the instrument operating in trapped ion mobility spectrometry (TIMS) mode. Trapped ions were selected for ms/ms using parallel accumulation serial fragmentation (PASEF). A scan range of (100–1700 m/z) was performed at a rate of 5 PASEF MS/MS frames to 1 MS scan with a cycle time of 1.03s^80^. The following chromatography buffers were used: Buffer B: 99.9% acetonitrile, 0.1% formic acid. Buffer A: 99.9% water, 0.1% formic acid. Raw mass spectrometric data have been deposited to the ProteomeXchange Consortium via the PRIDE^81^ partner repository with the dataset identifier PXD044594.

### Analysis of proteomics data

The raw data were searched against the Homo sapiens subset of the UniProt Swissprot database (reviewed, 12.11.2021) using the search engine Maxquant (release 2.1.4.0)^82^ with specific parameters for trapped ion mobility spectra data dependent acquisition (TIMS DDA). Each peptide used for protein identification met specific Maxquant parameters, i.e., only peptide scores that corresponded to a false discovery rate (FDR) of 0.01 were accepted from the Maxquant database search. The normalized protein intensity of each identified protein was used for label-free quantitation (LFQ)^83^. LFQ intensities were analyzed according to the protocol of Tyanova & Cox^37^, using R^74^. Proteins that were labelled as *reversed* or *potential contaminants* or *only identified by site* were excluded (see supplementary material). Proteins that were detected in less than half of the samples in all groups were also excluded. Every other protein was defined as identified and processed for quantiative analysis. Data were log2 transformed and the missing values were imputed with normal distribution around the detection limit. For statistical analysis, log2 transformed data were used. To calculate fold changes, non-transformed data was used. STRING software^84,85^ was used to visualize protein interaction networks and for gene ontology (GO) enrichment analysis.

### Monocyte activation assay

THP1 human monocytes expressing apoptosis-associated speck-like protein containing a CARD domain fused by green fluorescent protein (ASC-GFP, InvivoGen, Toulouse, France) were maintained in THP1 medium consisting of RPMI 1640 medium (Gibco, 21875-034), 10 V/V% heat inactivated FBS (Corning, 35-079-CV), 1% L-glutamine (Corning, 30-004-CI), 1% antibiotics-antimycotics (Corning, 25-005-CI), and 1% HEPES (Gibco, 15,-630-080) at a maximum of 6 × 10^5^ cells/mL in a T175 flask. THP1 medium was also supplemented with 100 μg/mL Zeocin (InvivoGen, ant-zn-0.5) for transgene selection at every second passage. Cells were grown at 5% CO2 95% air, at 37 °C. All experiments were performed within 10 passages and repeated at least four times.

1 × 10^6^ THP1-ASC-GFP cells in 24-well plates were treated with either 100 ng/mL LPS, 10^7^–10^9^ particles/mL HC-EV, CTRL-EV or VEH-EV, corresponding EV supernatants, or whole medium from AC16 cells overnight, and then cells were collected onto ice for flow cytometry analyses.

Cells were resuspended in PBS and fixed with 1% PFA at 4 °C for 10 min and washed twice. Flow cytometry was performed using BD FACSCalibur (BD Biosciences, San Jose, CA, USA) and evaluated using Flowing software (Turku Bioscience, Turku, Finland). THP1-ASC-GFP cells were gated first on live cells based on SSC and FSC followed by GFP^+^ population analysis. For each measurement, 10,000 events were counted.

### Quantitative PCR

After treatment of THP-1-ASC-GFP cells as described above, cells were lyzed in quiazol lysis reagent (Quiagen, Germantown, MD, USA) and stored at − 80 °C. Total RNA isolation, cDNA synthesis and qPCR measurements were performed as described earlier^86^. Measurements were performed on a LightCycler 480 Real-Time PCR System (Roche Diagnostics, Basel, Switzerland) using LightCycler^®^ RNA Master SYBR Green I reagent (Roche Diagnostics, Basel, Switzerland) with primers presented in Supplementary Table S2, with the following protocol: Initiation: 95 °C 2 min; Amplification: (45x) 95 °C 5 s, 57 °C 10 s, 72 °C 20 s. Data were analyzed by ΔΔCt calculation method according to Schmittgen and Livak 2008^87^, using Hypoxanthine Phosphoribosyltransferase 1 (HPRT) as a housekeeping gene.

### Statistics

For statistical analysis and data visualization, the programming language R with ggplot2^74,88^ was used. To create figures, Adobe Illustrator (Adobe, San Jose, CA, USA) was used. For statistical analysis, corresponding parametric statistical probes were applied with a significance level of 0.05. In more detail, multiple t-tests with Benjamini–Hochberg false discovery rate (FDR) adjustment were used for metabolomics data. To analyze AC16 EVs, including NTA, AFM and protein concentration data, and for the analysis of flow cytometry data on THP1-ASC-GFP cells, analysis of variance (ANOVA) with Tukey’s post-hoc test was applied. ANOVA with FDR adjustment followed by Tukey’s post-hoc test was used to analyze proteomics data. For GO enrichment analysis with FDR correction, STRING software was used^84,85^. Data are presented as mean ± standard error of the mean.

### Ethics approval

All procedures were approved by the National Scientific Ethical Committee on Animal Experimentation and the Semmelweis University’s Institutional Animal Care and Use Committee (H-1089 Budapest, Hungary) in accordance with NIH guidelines (National Research Council (2011), Guide for the Care and Use of Laboratory Animals: Eighth Edition) and permitted by the government of Food Chain Safety and Animal Health Directorate of the Government Office for Pest County (project identification code: PE/EA/1912-7/2017; date of approval: November 2017).

### Supplementary Information

Supplementary Information 1.

Supplementary Information 2.

Supplementary Information 3.

## Data Availability

Proteomic mass spectrometry data are available via ProteomeXchange with identifier PXD044594. Processed proteomic and metabolomic data, with the analyzed relative expression data are available as online supplementary material. All other datasets are available from the corresponding author on reasonable request.
