# Supplementary material for: Effect of hypercholesterolemia on circulating and cardiomyocyte-derived extracellular vesicles
Source: Sci Rep. 2024 May 26;14:12016. doi: 10.1038/s41598-024-62689-6 (PMC11128454; doi:10.1038/s41598-024-62689-6)
Supplement: Supplementary file 1 — Supplementary Information 1. [file 41598_2024_62689_MOESM1_ESM.docx]

Effect of hypercholesterolemia on circulating and cardiomyocyte-derived extracellular vesicles

Csenger Kovácsházi*^,1^, Szabolcs Hambalkó*^,1^, Nabil V. Sayour^1^, Tamás G. Gergely^1^, Gábor B. Brenner^1^, Csilla Pelyhe^1^, Dóra Kapui^1^, Bennet Y. Weber^1^, Alexander L. Hültenschmidt^1^, Éva Pállinger^2^, Edit I Buzás^2,3,4^, Ádám Zolcsák^5^, Bálint Kiss^5^, Tamás Bozó^5^, Csilla Csányi^5^, Nikolett Kósa^5^, Miklós Kellermayer^5^, Róbert Farkas^6^, Gellért B Karvaly^6^, Kieran Wynne^7^, David Matallanas^7^, Péter Ferdinandy^1,8^, Zoltán Giricz^1,6,†^

^1^ Semmelweis University, Department of Pharmacology and Pharmacotherapy, Budapest, Hungary

^2^ Semmelweis University, Department of Genetics, Cell- and Immunobiology, Budapest, Hungary

^3^ELKH-SE Translational Extracellular Vesicle Research Group, Budapest, Hungary

^4^ HCEMM-SU Extracellular Vesicle Research Group, Budapest, Hungary

^5^ Semmelweis University, Department of Biophysics and Radiation Biology, Budapest, Hungary

^6^ Semmelweis University, Department of Laboratory Medicine, Laboratory of Mass Spectrometry and Separation Technology, Budapest, Hungary

^7^ University College Dublin, Systems Biology Ireland and School of Medicine

^8^ Pharmahungary Group, Szeged, Hungary

* These authors contributed equally

^†^ Corresponding author <[giricz.zoltan@med.semmelweis-univ.hu](mailto:giricz.zoltan@med.semmelweis-univ.hu)>

## Detailed methodology of in vivo metabolomics measurement:

0.01 mL of EV sample, undiluted plasma, calibrator, or quality control (QC) samples were transferred to one of the wells of a 96-well plate of the MxP Quant 500 kit. Samples were dried with nitrogen (purity grade: 5.0) using positive pressure. Next, 0.05 mL of PITC solution (5% in a 1:1:1 v/v mixture of ethanol, pyridine and water) was added, and the deep well plate was allowed to stand at room temperature (60 min). After evaporation of the solvent under a stream of nitrogen, 0.3 mL of ammonium-acetate (5 mmol/L in methanol) was pipetted into each slot, and the plate was kept on orbital shaker for 30 min. The liquid contents of the deep well plate were forced onto a collection plate by applying positive pressure, and 0.15 mL of these eluates were pipetted onto another collection plate for Runs 1 and 2, and diluted with 0.15 mL of water. 0.01 mL of eluate was transferred onto yet another collection plate for Runs 3 and 4, and diluted using 0.49 mL of flow injection analysis mobile phase. The analysis was conducted using a modular system containing a Shimadzu XR liquid chromatograph (DGU-20 degasser, LC-20AD quaternary pump, SIL-20AC autosampler, CTO-40AC column thermostat, Simkon Kft., Budapest, Hungary) and an AB Sciex 5500QTRAP triple quadrupole linear ion trap mass spectrometer (Per-Form Hungária Kft., Budapest, Hungary). The analytical methods provided with the metabolomic reagent kit were used. The stationary phase, kept at 50 °C, was supplied with the kit. The mobile phases employed for Runs 1 and 2 were water and acetonitrile, both containing 0.2% formic acid. For the flow injection analysis (Runs 3 and 4), the employed mobile phase was 290 mL of methanol containing 1 ampule of FIA Mobile Phase Additive provided with the reagent kit. The mass spectrometer was operated in multiple reaction monitoring mode. Four analytical runs were conducted with the settings detailed in Tables 1 and 3. The volume of the sample injected was 5 µL in Runs 1 and 2, and 20 µL in Runs 3 and 4. Analyte-specific mass spectrometry settings were provided by the kit manufacturer.

Six-point calibrations of 42 components were performed using linear regression with 1/x weights, except for histamine (linear regression, no weights), arginine, and creatinine (quadratic regression, no weights), as well as dihydroxyphenylalanine (DOPA) and dopamine (quadratic regression, 1/x weights). Single-level calibration was performed for 588 analytes using Equation (S1), as recommended by the kit manufacturer:

$c_{analyte}=\frac{{Peak area}_{analyte}\cdot c_{internal standard}}{{Peak area}_{internal standard}}$ (S1)

where c_analyte_ is the concentration of the analyte, and c_internal standard_ is the known concentration of the internal standard in the mixture obtained by adding the 5% PITC solution to the dried extracts.

QC results were evaluated by the MetIDQ software. Level 1 and level 3 samples were run once, while the QC level 2 sample was pipetted into positions 17, 22, 50, 67, and 96 of the 96-well plate, as recommended by the kit manufacturer in order to evaluate the reproducibility of measurements.

## Supplementary figures:


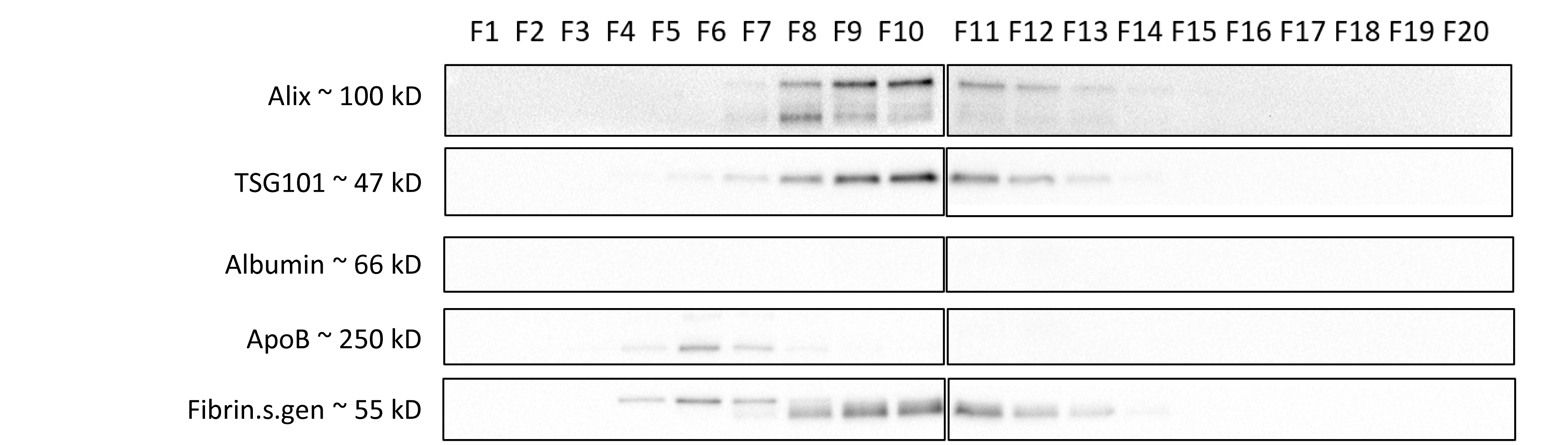


Supplementary Figure S1: Representative image of western blot analysis of plasma samples purified with density gradient ultracentrifugation followed by size exclusion chromatography. Fractions F8-10 were combined and used as plasma EV isolates.


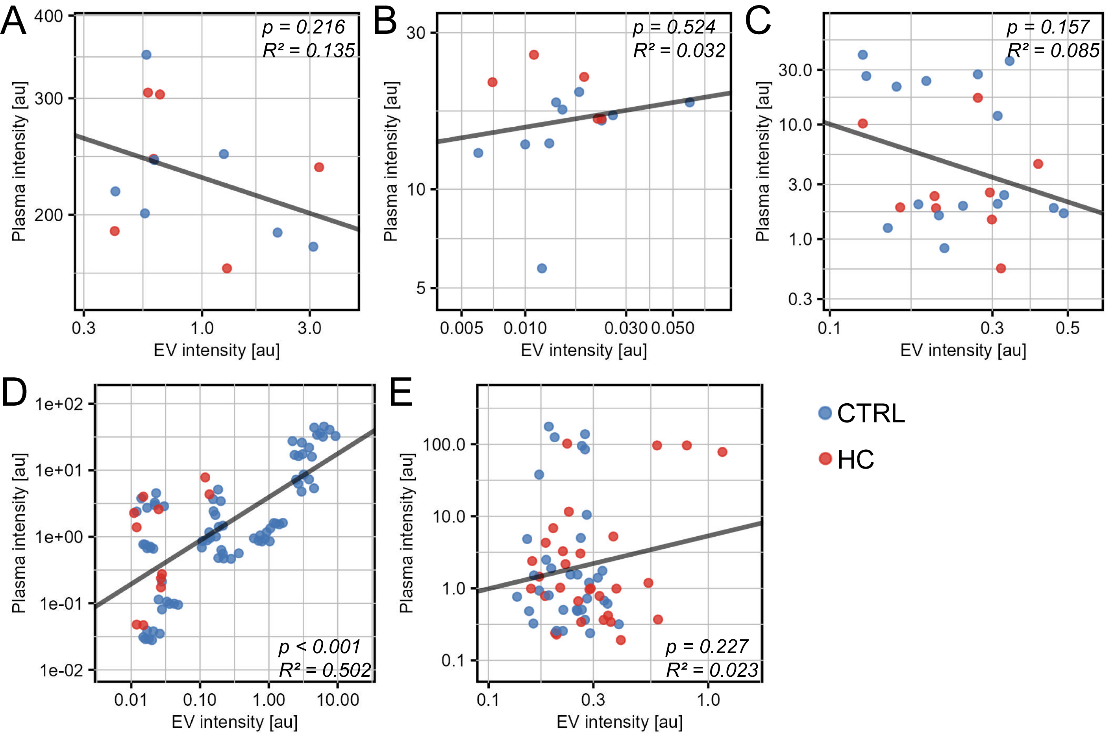


Supplementary Figure S2: Correlation analysis between intensities of metabolites detected in both plasma and in EVs separated by metabolite groups. A: Aminoacids, B: Aminoacids related, C: Fatty acids, D: glycerophospholipids, E: triacylglycerols. Highlighted p-value and R-squared represent the results of linear regression analyses. (n_ctrl_ = 11, n_hc_ = 7 for all experiments)


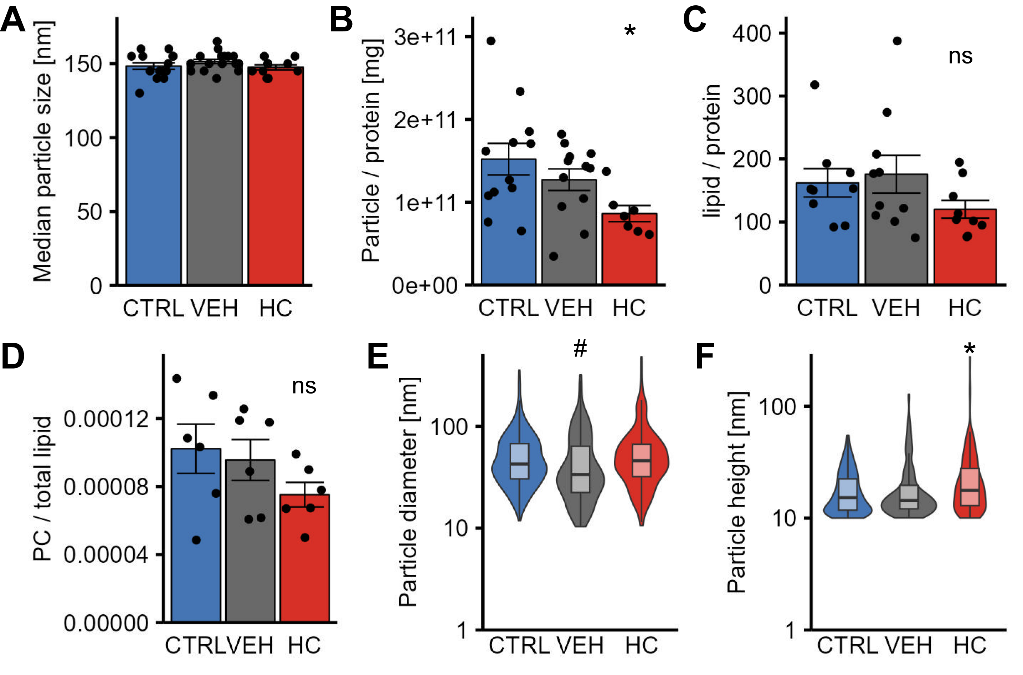


Supplementary Figure S3: Detailed analysis of AC16 EVs. A: Median size of isolated particles obtained by NTA. No difference was observed. ( n_ctrl_ = 15, n_veh_ = 17, n_hc_ = 9) B: Particle/protein ratio decreased in HC (n_ctrl_ = 12, n_veh_ = 12, n_hc_ = 7) C: No statistically significant difference was observed in lipid/protein ratio (n_ctrl_ = 9, n_veh_ = 10, n_hc_ = 9) D: Analysis of phosphatidylcholine (PC)/lipid content of AC16 EVs. No significant difference was observed. (n = 6 all groups) E: Particle diameter obtained from AFM measurements. A slight, however, significant decrease in the VEH group versus the other groups was observed. (n_ctrl_ = 288, n_veh_ = 232, n_hc_ = 212 out of at least three independent experiments) F: Particle height obtained from AFM measurements. A slight, however, significant increase in HC versus the other groups was observed. (n_ctrl_ = 288, n_veh_ = 232, n_hc_ = 212 out of at least three independent experiments) * p < 0.05 HC vs CTRL and VEH; # p < 0.05 VEH vs CTRL and HC; ns p > 0.2 ANOVA with Tukey’s post-hoc test.


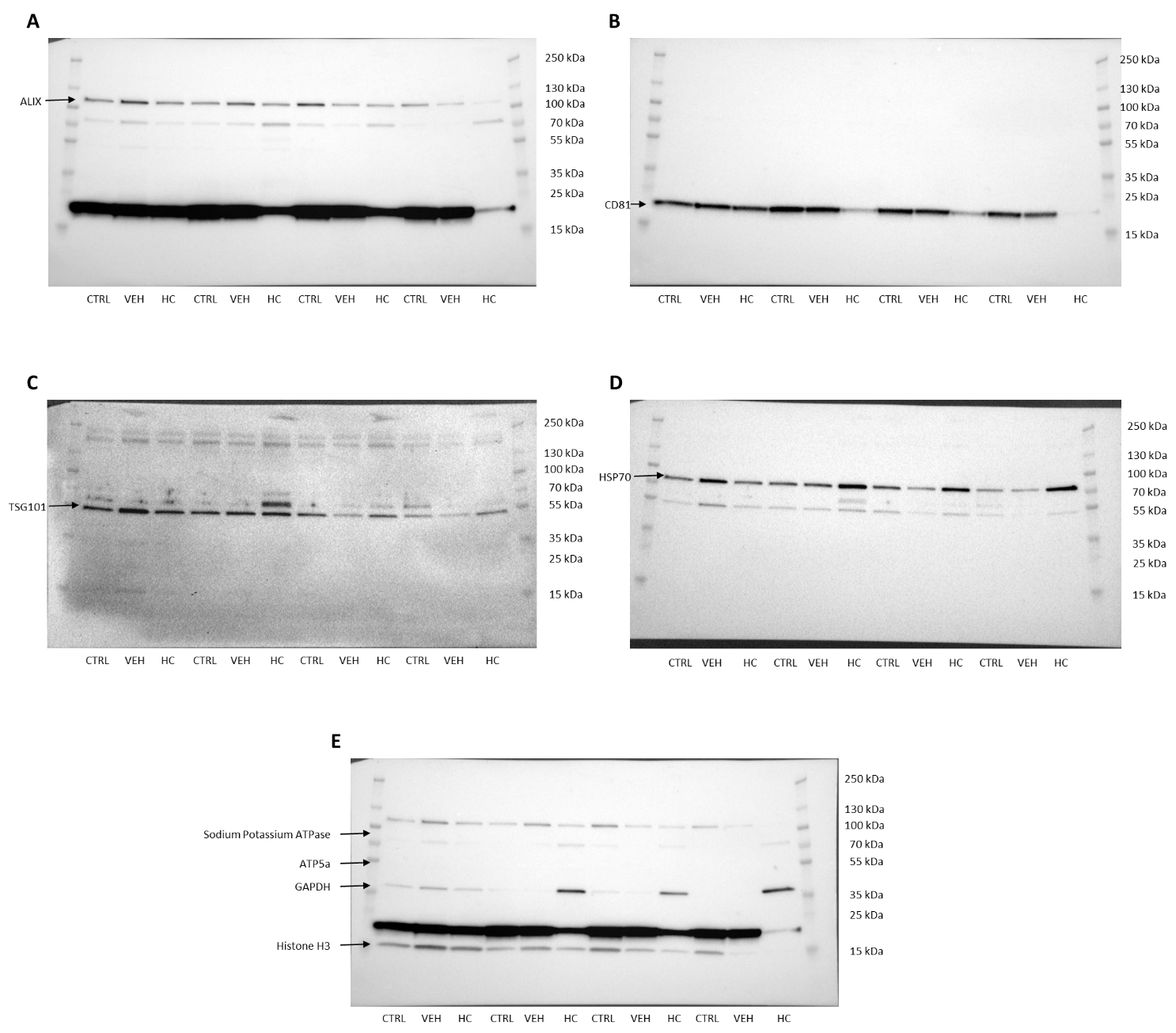


Supplementary Figure S4: Whole western blot images of (**A**) ALIX, (**B**) CD81, (**C**) TSG101, (**D**) HSP70**,** (**E**) Sodium Potassium ATPase, ATP5A, Histone H3 and GAPDH of AC16 EVs.


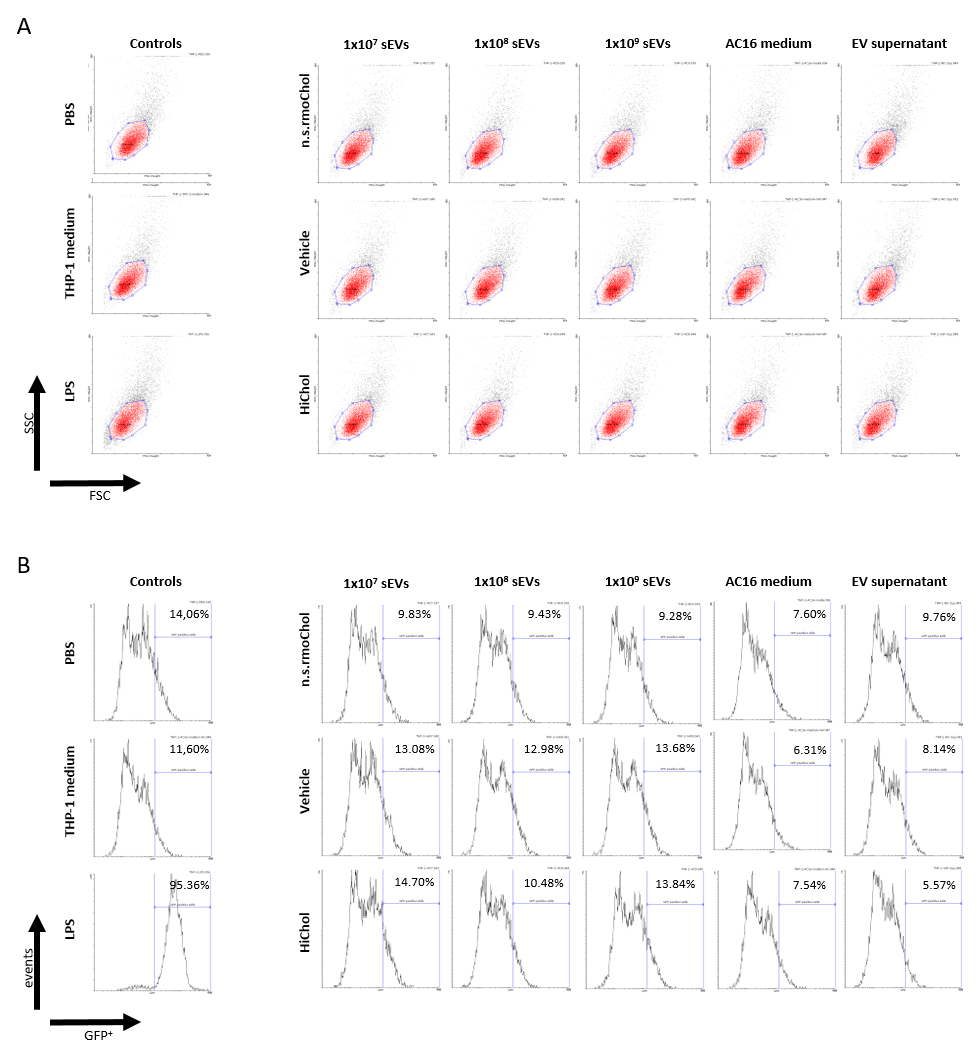


Supplementary Figure S5: Representative images of flow cytometry data on GFP expression in THP1-ASC-GFP cells. A: Gating of viable single cells. B: Graphs depicting representative histograms illustrating the percentage of cells expressing GFP under various treatment conditions.

## Supplementary tables:

Supplementary Table S1: Details of the employed gradient liquid chromatographic program (Runs 1 and 2).

| **Run ID** | **Time (min)** | **Flow rate (mL/min)** | **Mobile phase A volumetric ratio (%)** | **Mobile phase B volumetric ratio (%)** |
| --- | --- | --- | --- | --- |
| 1 | 0.0 | 0.5 | 100 | 0.0 |
|  | 0.25 | 0.5 | 100 | 0.0 |
|  | 1.0 | 0.5 | 88.0 | 12.0 |
|  | 3.0 | 0.5 | 82.5 | 17.5 |
|  | 4.5 | 0.5 | 50.0 | 50.0 |
|  | 5.5 | 0.5 | 0.0 | 100 |
|  | 7.0 | 0.6 | 0.0 | 100 |
|  | 7.5 | 0.8 | 0.0 | 100 |
|  | 7.6 | 0.8 | 100 | 0.0 |
|  | 8.5 | 0.8 | 100 | 0.0 |
|  | 9.5 | 0.5 | 100 | 0.0 |
|  | 11.0 | 0.5 | 100 | 0.0 |
| 2 | 0.0 | 0.5 | 100 | 0.0 |
|  | 0.25 | 0.5 | 100 | 0.0 |
|  | 0.5 | 0.5 | 75.0 | 25.0 |
|  | 3.0 | 0.5 | 50.0 | 50.0 |
|  | 4.0 | 0.5 | 25.0 | 75.0 |
|  | 4.5 | 0.5 | 0.0 | 100 |
|  | 6.5 | 0.7 | 0.0 | 100 |
|  | 7.5 | 0.8 | 0.0 | 100 |
|  | 7.6 | 0.8 | 100 | 0.0 |
|  | 8.5 | 0.8 | 100 | 0.0 |
|  | 9.5 | 0.5 | 100 | 0.0 |
|  | 11.0 | 0.5 | 100 | 0.0 |

Supplementary Table S2: Primers used in THP-1-ASC-GFP activation assay

| **Gene name** | **Gene abbreviation** | **Primers sequence** |
| --- | --- | --- |
| Interleukin 1 Beta | IL1b | ATTGCTCAAGTGTCTGAAGCAGC |
|  |  | AGCCCTTGCTGTAGTGGTGG |
| Interleukin 10 | IL-10 | GCCTTCAGCAGAGTGAAGACTTTCTTTC |
|  |  | CCAGGTAAAACTGGATCATCTCAGACAAGGC |
| Hypoxanthine Phosphoribosyltransferase 1 | HPRT | TGCTCGAGATGTGATGAAGG |
|  |  | TCCCCTGTTGACTGGTCATT |
| Tumor Necrosis Factor-Alpha | TNFa | ATACTGACCCACGGCTCCAC |
|  |  | AGAAGATGATCTGACTGCCTGGG |
| Interleukin 6 | IL6 | GGTACATCCTCGACGGCATCT |
|  |  | GTGCCTCTTTGCTGCTTTCAC |

Supplementary Table S3: General mass spectrometry settings for metabolomics analysis of plasma and plasma EVs

| **Run ID** | **Stationary phase** | **ionization polarity** | **Curtain gas (L/min)** | **Collision gas (L/min)** | **Ion spray voltage (V)** | **Ion source temperature (°C)** | **Ion source gas 1 (L/min)** | **Ion source gas 2 (L/min)** |
| --- | --- | --- | --- | --- | --- | --- | --- | --- |
| 1 | yes | positive | 45 | 9 | 5500 | 500 | 60 | 70 |
| 2 | yes | negative | 20 | 8 | -4500 | 650 | 40 | 40 |
| 3 | none | positive | 20 | 9 | 5500 | 200 | 40 | 50 |
| 4 | none | positive | 10 | 9 | 5500 | 350 | 30 | 90 |

Supplementary Table S4: Differentially expressed proteins in AC16 EVs. Genes which were significant between VEH and HC were used for further analysis. (n_ctrl_ = 10, n_veh_ = 10, n_hc_ = 4) * p < 0.05 for ANOVA followed by Tukey’s post-hoc test.

| **#** | **Protein IDs** | **Gene names** | **ANOVA Post-hoc significance** | | | **Log2 fold change** | | |
| --- | --- | --- | --- | --- | --- | --- | --- | --- |
|  |  |  | **CTRL vs VEH** | **CTRL vs HC** | **VEH vs HC** | **CTRL vs VEH** | **CTRL vs HC** | **VEH vs HC** |
| 1 | O14672;O14672-2 | ADAM10 | * | ns | * | -0.323 | -1.762 | -1.439 |
| 2 | P25098;P35626 | ADRBK1;ADRBK2 | * | * | ns | 3.492 | 4.442 | 0.950 |
| 3 | Q02952-3;Q02952-2;Q02952 | AKAP12 | * | ns | * | -0.163 | -1.324 | -1.161 |
| 4 | Q86V81 | ALYREF | * | * | * | 1.878 | 4.210 | 2.332 |
| 5 | O43633 | CHMP2A | * | ns | * | 0.718 | -2.066 | -2.784 |
| 6 | Q9NZZ3;Q9NZZ3-2 | CHMP5 | * | ns | * | -0.231 | -1.695 | -1.464 |
| 7 | Q9Y3Y2-4;Q9Y3Y2;Q9Y3Y2-3 | CHTOP | * | ns | * | 0.558 | 3.423 | 2.864 |
| 8 | Q99715;Q99715-4;Q99715-2 | COL12A1 | * | * | * | -0.540 | -1.581 | -1.041 |
| 9 | P12111;P12111-2;P12111-4; P12111-5;P12111-3 | COL6A3 | * | * | ns | -0.380 | -0.950 | -0.570 |
| 10 | P29279;P29279-2 | CTGF | * | ns | * | -0.595 | 2.859 | 3.454 |
| 11 | O00622 | CYR61 | * | ns | * | -0.107 | 1.719 | 1.826 |
| 12 | Q6UVK1 | CSPG4 | * | ns | ns | -0.357 | -0.929 | -0.572 |
| 13 | Q5VWQ8-3;Q5VWQ8-4;Q5VWQ8-2; Q5VWQ8-5;Q9UJF2; Q5VWQ8;Q9UJF2-2 | DAB2IP;RASAL2 | * | ns | * | -0.916 | 1.236 | 2.152 |
| 14 | Q92499;Q92499-3;Q92499-2 | DDX1 | * | ns | * | 0.050 | 1.250 | 1.200 |
| 15 | Q92841;Q92841-1;Q92841-3; Q92841-2 | DDX17 | * | ns | * | 0.367 | 1.431 | 1.064 |
| 16 | Q9NR30;Q9NR30-2;Q9BQ39 | DDX21 | * | ns | * | 0.272 | 3.135 | 2.863 |
| 17 | Q9P265 | DIP2B | * | ns | * | 0.273 | -3.455 | -3.728 |
| 18 | P26358-2;P26358;P26358-3 | DNMT1 | * | ns | * | -0.449 | 3.174 | 3.624 |
| 19 | O43854-2;O43854 | EDIL3 | * | ns | * | -0.773 | -1.820 | -1.046 |
| 20 | P26641;P26641-2 | EEF1G | * | * | ns | 0.402 | 0.755 | 0.353 |
| 21 | O00303 | EIF3F | * | ns | ns | 0.567 | 1.296 | 0.729 |
| 22 | Q9Y6C2;Q9Y6C2-2 | EMILIN1 | * | ns | * | -0.036 | -1.501 | -1.465 |
| 23 | Q52LJ0-1;Q52LJ0 | FAM98B | * | ns | * | 0.566 | 2.812 | 2.246 |
| 24 | P62942 | FKBP1A | * | ns | * | 0.214 | -2.979 | -3.194 |
| 25 | P35637-2;P35637 | FUS | * | ns | * | 0.227 | 1.378 | 1.151 |
| 26 | P51114;P51114-3;P51114-2 | FXR1 | * | ns | * | 0.531 | 2.572 | 2.041 |
| 27 | P51116 | FXR2 | * | ns | * | 0.684 | 3.722 | 3.038 |
| 28 | P17302 | GJA1 | * | ns | * | -0.818 | -3.718 | -2.900 |
| 29 | Q92896;Q92896-3;Q92896-2 | GLG1 | * | ns | * | -0.224 | -1.125 | -0.900 |
| 30 | P63244 | GNB2L1 | * | ** | * | 0.631 | 1.189 | 0.557 |
| 31 | P29084 | GTF2E2 | * | ns | * | 0.938 | 4.489 | 3.550 |
| 32 | Q8IUE6;Q71UI9-5 | HIST2H2AB | * | ns | * | 0.389 | -2.047 | -2.437 |
| 33 | P01889 | HLA-B | * | ns | * | -0.444 | -2.192 | -1.748 |
| 34 | Q13151 | HNRNPA0 | * | ns | * | 0.890 | 3.425 | 2.535 |
| 35 | P09651-3;P09651-2;P09651;Q32P51 | HNRNPA1;HNRNPA1L2 | * | ns | * | 0.204 | 1.009 | 0.805 |

*Supplementary Table S4 continued*

| **#** | **Protein IDs** | **Gene names** | **ANOVA Post-hoc significance** | | | **Log2 fold change** | | |
| --- | --- | --- | --- | --- | --- | --- | --- | --- |
|  |  |  | **CTRL vs VEH** | **CTRL vs HC** | **VEH vs HC** | **CTRL vs VEH** | **CTRL vs HC** | **VEH vs HC** |
| 36 | P22626;P22626-2 | HNRNPA2B1 | * | ns | * | 0.169 | 0.761 | 0.592 |
| 37 | P51991 | HNRNPA3 | * | ns | * | -0.026 | 0.990 | 1.016 |
| 38 | Q14103-3;Q14103;Q14103-4; Q14103-2 | HNRNPD | * | * | * | 0.348 | 0.991 | 0.642 |
| 39 | O43390;O43390-3 | HNRNPR | * | ns | * | 0.203 | 1.845 | 1.642 |
| 40 | Q5SSJ5;Q5SSJ5-3;Q5SSJ5-2; Q5SSJ5-5 | HP1BP3 | * | ns | * | 0.392 | -1.463 | -1.855 |
| 41 | Q0VDF9 | HSPA14 | * | ns | * | 0.490 | 2.277 | 1.787 |
| 42 | P98160 | HSPG2 | * | ns | * | -0.136 | -1.221 | -1.085 |
| 43 | Q16666-3;Q16666-2;Q16666; Q16666-6;Q6K0P9-6;Q6K0P9-5; Q6K0P9-4;Q6K0P9-3;Q6K0P9-2; Q6K0P9 | IFI16 | * | ns | * | -0.400 | 3.619 | 4.020 |
| 44 | Q9NZI8;Q9NZI8-2 | IGF2BP1 | * | ns | * | -0.557 | 3.187 | 3.744 |
| 45 | Q9Y6M1-1;Q9Y6M1;Q9Y6M1-5; Q9Y6M1-6;Q9Y6M1-3;Q9Y6M1-4 | IGF2BP2 | * | ns | * | -0.332 | 2.963 | 3.296 |
| 46 | Q969P0;Q969P0-3;Q969P0-2 | IGSF8 | * | ns | * | 0.303 | -1.213 | -1.516 |
| 47 | P53990-2;P53990-3;P53990; P53990-4;P53990-5 | IST1 | * | * | ns | -0.869 | -1.979 | -1.110 |
| 48 | Q6NYC1-2;Q6NYC1;Q6NYC1-3 | JMJD6 | * | ns | * | -0.343 | 1.845 | 2.188 |
| 49 | Q16363-2;Q16363 | LAMA4 | * | ns | * | -0.334 | -0.888 | -0.554 |
| 50 | Q6PKG0;Q6PKG0-3;Q659C4-7; Q659C4-6;Q659C4-5;Q659C4-2; Q659C4;Q659C4-9 | LARP1 | * | ns | * | 0.657 | 3.122 | 2.465 |
| 51 | Q4G0J3-3;Q4G0J3;Q4G0J3-2 | LARP7 | * | ns | * | 0.183 | 3.079 | 2.896 |
| 52 | P09382 | LGALS1 | * | ns | * | -0.235 | -1.297 | -1.062 |
| 53 | Q9NX58 | LYAR | * | ns | * | 0.620 | 4.227 | 3.607 |
| 54 | Q9UNF1-2;Q9UNF1;Q12816-5; Q12816-2;Q12816-4;Q12816 | MAGED2 | * | ns | * | 1.430 | 3.711 | 2.281 |
| 55 | Q7Z304 | MAMDC2 | * | ns | * | -0.173 | -2.661 | -2.488 |
| 56 | P29966 | MARCKS | * | ns | * | -0.247 | -1.399 | -1.152 |
| 57 | Q08431;Q08431-3;Q08431-4; Q08431-2 | MFGE8 | * | ns | * | -0.275 | -1.298 | -1.023 |
| 58 | Q9HCE1;Q9HCE1-2 | MOV10 | * | ns | * | -0.353 | 1.751 | 2.104 |
| 59 | P19338 | NCL | * | ns | * | -0.067 | 2.023 | 2.089 |
| 60 | Q14112-2;Q14112 | NID2 | * | ns | * | -0.255 | -1.501 | -1.247 |
| 61 | Q8TAD7 | OCC1 | * | ns | * | -0.218 | -1.280 | -1.061 |
| 62 | P11940;P11940-2;Q9H361;Q4VXU2; Q4VXU2-2;Q96DU9-2;Q96DU9; Q5JQF8 | PABPC1 | * | ns | * | 0.292 | 1.328 | 1.035 |
| 63 | Q13310-2;Q13310;Q13310-3 | PABPC4 | * | ns | * | 0.021 | 1.625 | 1.604 |
| 64 | P09874 | PARP1 | * | ns | * | 0.269 | 1.497 | 1.228 |
| 65 | Q8WUM4-2;Q8WUM4;Q8WUM4-3 | PDCD6IP | * | ns | * | -0.223 | -1.054 | -0.831 |
| 66 | P30086 | PEBP1 | * | ns | * | -0.048 | -1.851 | -1.804 |
| 67 | Q8IWS0;Q8IWS0-5;Q8IWS0-3; Q8IWS0-4;Q8IWS0-2 | PHF6 | * | ns | * | -0.449 | 4.134 | 4.583 |
| 68 | O15031 | PLXNB2 | * | ns | * | -0.542 | -1.175 | -0.634 |
| 69 | Q9NRX1 | PNO1 | * | ns | * | 1.221 | 3.484 | 2.264 |
| 70 | P48634-2;P48634-3;P48634;P48634-4 | PRRC2A | * | ns | * | -0.897 | 3.855 | 4.752 |
| 71 | Q9Y520-3;Q9Y520-4;Q9Y520-5; Q9Y520;Q9Y520-7;Q9Y520-2; Q9Y520-6 | PRRC2C | * | ns | * | -0.260 | 3.920 | 4.180 |
| 72 | O95084-2;O95084 | PRSS23 | * | ns | * | -0.270 | -1.564 | -1.295 |
| 73 | O75475;O75475-3;O75475-2 | PSIP1 | * | ns | * | 0.009 | 3.230 | 3.221 |
| 74 | Q8IY67-2;Q8IY67;Q8IY67-3 | RAVER1 | * | ns | * | -0.587 | 2.153 | 2.739 |

*Supplementary Table S4 continued*

| **#** | **Protein IDs** | **Gene names** | **ANOVA Post-hoc significance** | | | **Log2 fold change** | | |
| --- | --- | --- | --- | --- | --- | --- | --- | --- |
|  |  |  | **CTRL vs VEH** | **CTRL vs HC** | **VEH vs HC** | **CTRL vs VEH** | **CTRL vs HC** | **VEH vs HC** |
| 75 | P38159;P38159-2;Q96E39; P38159-3;O75526;Q8N7X1 | RBMX;RBMXL1 | * | ** | * | 0.906 | 2.826 | 1.920 |
| 76 | P62906 | RPL10A | * | ns | * | 0.300 | 1.820 | 1.520 |
| 77 | P18621-3;P18621;P18621-2 | RPL17 | * | ns | * | 0.131 | 1.629 | 1.497 |
| 78 | Q07020-2;Q07020 | RPL18 | * | ns | * | 0.217 | 1.532 | 1.316 |
| 79 | P84098 | RPL19 | * | ns | * | 0.081 | 1.816 | 1.736 |
| 80 | P46778 | RPL21 | * | ns | * | 0.247 | 1.390 | 1.143 |
| 81 | P61353 | RPL27 | * | ns | * | 0.246 | 1.315 | 1.069 |
| 82 | P62888 | RPL30 | * | ns | * | 0.474 | 1.412 | 0.938 |
| 83 | P62910 | RPL32 | * | ns | * | 0.294 | 1.552 | 1.258 |
| 84 | P05387;P05386-2 | RPLP2 | * | ns | * | 0.264 | 1.010 | 0.746 |
| 85 | P46783;Q9NQ39 | RPS10;RPS10P5 | * | ns | * | 0.292 | 1.618 | 1.326 |
| 86 | P62280 | RPS11 | * | ns | * | 0.076 | 1.168 | 1.092 |
| 87 | P25398 | RPS12 | * | ns | * | 0.282 | 1.483 | 1.202 |
| 88 | P62263 | RPS14 | * | ns | * | 0.490 | 2.135 | 1.645 |
| 89 | P62841 | RPS15 | * | ns | * | 0.102 | 1.724 | 1.622 |
| 90 | P62249 | RPS16 | * | ns | * | 0.474 | 1.534 | 1.060 |
| 91 | P15880 | RPS2 | * | ns | * | 0.090 | 1.153 | 1.062 |
| 92 | P60866;P60866-2 | RPS20 | * | ns | * | 0.002 | 1.631 | 1.629 |
| 93 | P62847-2;P62847-3;P62847;P62847-4 | RPS24 | * | * | * | 0.635 | 2.067 | 1.432 |
| 94 | P62854;Q5JNZ5 | RPS26;RPS26P11 | * | ns | * | 0.258 | 1.665 | 1.407 |
| 95 | P61247 | RPS3A | * | ns | * | 0.300 | 1.505 | 1.205 |
| 96 | P46782 | RPS5 | * | ns | * | 0.235 | 1.634 | 1.399 |
| 97 | P08865 | RPSA | * | ns | * | 0.205 | 1.116 | 0.910 |
| 98 | Q9P2E9;Q9P2E9-3;Q8N4C6-6; Q8N4C6-11 | RRBP1 | * | ns | * | -0.321 | 1.021 | 1.343 |
| 99 | O43290 | SART1 | * | ns | * | -0.865 | 2.419 | 3.284 |
| 100 | Q99590-2;Q99590 | SCAF11 | * | ns | * | -0.198 | 2.483 | 2.681 |
| 101 | O00560-2 | SDCBP | * | ns | * | -0.061 | -1.341 | -1.280 |
| 102 | P34897-3;P34897;P34897-2 | SHMT2 | * | ns | * | -0.095 | -1.960 | -1.864 |
| 103 | Q9NUQ6;Q9NUQ6-2;Q9NUQ6-4; Q9NUQ6-3 | SPATS2L | * | ns | * | -0.478 | 3.162 | 3.639 |
| 104 | P37108 | SRP14 | * | ns | * | -0.187 | 1.316 | 1.503 |
| 105 | Q08945 | SSRP1 | * | ns | * | 0.717 | 2.184 | 1.467 |
| 106 | O95793-2;O95793;O95793-3 | STAU1 | * | ns | * | 0.610 | 2.728 | 2.118 |
| 107 | O60506-3;O60506;O60506-4; O60506-2;O60506-5 | SYNCRIP | * | * | * | 0.279 | 1.283 | 1.004 |
| 108 | P35442 | THBS2 | * | ns | * | -0.393 | -1.363 | -0.970 |
| 109 | Q96EY4 | TMA16 | * | ns | * | -2.328 | 1.574 | 3.902 |
| 110 | P62328 | TMSB4X | * | ns | * | -0.110 | -1.232 | -1.122 |
| 111 | Q99816;Q99816-2 | TSG101 | * | * | ns | -0.764 | -1.378 | -0.614 |
| 112 | O43657 | TSPAN6 | * | ns | * | -0.150 | -1.767 | -1.617 |
| 113 | Q9C0H2-3;Q9C0H2-2;Q9C0H2; Q9C0H2-4 | TTYH3 | * | ns | * | -0.530 | -4.459 | -3.928 |
| 114 | P67809;Q9Y2T7 | YBX1 | * | ns | * | 0.337 | 1.337 | 1.000 |
| 115 | P16989-2 | YBX3 | * | ns | * | 1.562 | 4.869 | 3.308 |
| 116 | P16989;P16989-3 | YBX3 | * | ns | * | 0.767 | 4.128 | 3.361 |
| 117 | Q6DD87 | ZNF787 | * | ns | * | 0.992 | 3.983 | 2.991 |
